# A full-spectrum *Boswellia serrata* extract with enhanced bioavailability, and its co-delivered system with curcumin alleviate pain and stiffness associated with moderate spondylitis: a randomized double-blind, placebo-controlled, 3-arm study

**DOI:** 10.3389/fphar.2025.1577429

**Published:** 2025-07-01

**Authors:** K. Mamatha, Prathibha Prabhakaran, S. Syam Das, Manu Kanjoormana Aryan, Jestin Thomas

**Affiliations:** ^1^ Department of General Medicine, Divakar’s Specialty Hospital, Bengaluru, Karnataka, India; ^2^ R&D Centre, Akay Natural Ingredients, Kochi, Kerala, India; ^3^ Department of Immunology, Amala Cancer Research Centre, Thrissur, Kerala, India; ^4^ Leads Clinical Research and Bio Services Private Limited, Bengaluru, Karnataka, India

**Keywords:** spondylitis, neck/back/joint pain, Curcumin, boswellia, bioavailability

## Abstract

**Objective:**

Pain and stiffness at the neck, back, hip, and other joints due to conditions such as spondylitis or spondylosis are significant musculoskeletal concerns globally. The present study evaluated the relative efficacy of a bioavailability enhanced full-spectrum extract of *Boswellia serrata Roxb*. oleo-gum resin (F-BSE) and its co-delivery system with curcumin (C-BSE) in ameliorating pain and stiffness, in otherwise healthy participants.

**Methods:**

This study adopted a randomized, double-blind, placebo-controlled, three-arm, parallel group comparative design. The participants received the placebo, F-BSE, or C-BSE for 28 days at the dose of 400 mg/day. The efficacy was assessed using subjective tools [Bath Ankylosing Spondylitis Disease Activity Index (BASDAI) and Neck Disability Index (NDI) questionnaires) and objective measures (clinical markers, NLRP3 and IL-1β).

**Results:**

A total of 105 participants (n = 35/group) were recruited, of which 94 completed the 28-day study period. Both the F-BSE and C-BSE groups showed significant reductions in pain, stiffness, and neck-related disability, as reflected in the BASDAI and NDI scores. Additionally, NLRP3 inflammasome and IL-1β levels were significantly reduced by days 14 and 28. These outcomes suggest that the combined administration of curcuminoids and boswellic acids with improved bioavailability modulates inflammatory pathways, thus contributing to symptom relief and improved function.

**Conclusion:**

Both F-BSE and C-BSE reduced pain and stiffness issues by day 14, which continued to progress through day 28. However, C-BSE demonstrated superior effects compared to F-BSE, indicating a synergistic anti-inflammatory and analgesic action when the full-spectrum Boswellia extract and curcumin are delivered as a water-soluble, non-covalent complex with enhanced bioavailability using the FenuMat^®^ delivery technology.

**Clinical trial registration:**

https://ctri.nic.in/Clinicaltrials/pmaindet2.php?EncHid=NjI2MTY=&Enc=&userName=, identifier: CTRI/2021/12/038613 dated 14/12/2021.

## 1 Introduction

Pain, an adverse sensory response triggered from either neuropathic (nerve-related damage) or nociceptive (tissue-related damage) origins, is a central hallmark of numerous clinical disorders. Inflammation and the immune system play a key role in progression and sensation of pain by activating sensory neurons (Starobova et al., 2020). Pain at the neck, back, hip, and other joints due to spondylitis or spondylosis is a significant musculoskeletal concern globally, and its severity may vary from mild to moderate to severe, leading to a significant impact on mental health and work disability (Webers et al., 2019). Joint stiffness limits mobility, and hence, physical movements. Insufficient physical activity, extended usage of computer and mobile phones in poor postures such as forward head positioning, infection, etc. have been generally shown to initiate and aggravate such conditions (Lee et al., 2025). With its rising prevalence in the United States, Western Europe, and East Asia, it is becoming increasingly important to explore the connections between neck/back/hip and other joint pains and spinal mobility with the quality of life (Chen et al., 2022; Safiri et al., 2020).

Inflammasomes are cytoplasmic protein complexes consisting of sensory proteins, caspases, and adapter proteins that activate the inflammatory response and induce pain (Starobova et al., 2020). They are innate immune system receptors/sensors and are triggered in response to exogenous or endogenous stimuli such as musculoskeletal wear and tear or infection, resulting in the secretion of pro-inflammatory cytokines (Zheng et al., 2020; de Zoete et al., 2014). Nucleotide-binding domain, leucine-rich-containing family, pyrin domain-containing-3 (NLRP3) is the most widely studied inflammasome involved in the onset and progression of inflammation. It plays an important role in innate and adaptive immune systems as well as in the development of conditions such as arthritis, spondylitis, spondylosis, and gout (Yang et al., 2022). NLRP3 is expressed mainly in blood leukocytes, and it can activate caspase 1 in response to an inflammatory stimulus, leading to the maturation of pro-inflammatory cytokines (Yang et al., 2022; Seok et al., 2021). IL-1β and IL-18 are important cytokines involved in the pathogenesis of joint inflammation, bone destruction, and breakdown of cartilages and ligaments, especially in the spine (van de Veerdonk et al., 2011). Bioactive molecules (synthetic or natural) that can inhibit or suppress the production of such inflammatory mediators, thus, may find therapeutic applications in the treatment of spondylitis.

Current treatment modalities for spondylitis and spondylosis mainly include pharmacological agents such as non-steroidal anti-inflammatory drugs (NSAIDs) and tumor necrosis factor inhibitors, which can suppress NLRP3 activity. Non-pharmacological practices such as regular exercise, postural training, and physical therapy were also suggested as first-line therapies, either alone or in combination with drugs, depending on the severity of the disease state and pain (Wenker and Quint, 2020; Feng et al., 2023). However, these drugs are accompanied by adverse effects such as nasopharyngitis, headache, nausea, gastrointestinal disorders, and injection-site reactions (Feng et al., 2023). Recently, various herbs and phytonutrients have been shown to exhibit significant anti-inflammatory and anti-nociceptive properties that are suitable for the management of mild-to-moderate pain (Lindler et al., 2020; Dragos et al., 2017).

*Boswellia serrata Roxb.* and *Curcuma longa L.* (turmeric) extracts, standardized with respect to their bioactive molecules, boswellic acids and curcuminoids, respectively, are widely used as nutraceuticals/dietary supplements for musculoskeletal pain and inflammation, mainly for osteoarthritis (Bannuru et al., 2018; Sethi et al., 2022). Preclinical studies have shown that these phytochemicals exhibit a synergistic action to alleviate inflammation and pain when administrated at a pharmacologically effective dosage (Sethi et al., 2022; Haroyan et al., 2018; Cardeccia et al., 2022). However, both curcuminoids and boswellic acids possess poor oral bioavailability, and hence, a high dosage (500–1,000 mg of curcumin along with 500 mg of Boswellia extracts twice daily) is generally recommended for clinical efficacy (Bannuru et al., 2018; Haroyan et al., 2018). We anticipated that the supplementation with bioavailability enhanced formulations of boswellic acids and curcumin would be beneficial for the management of pain and stiffness issues associated with spondylitis/spondylosis conditions.

Recently, natural self-emulsifying reversible hybrid-hydrogel (N’SERH) formulations of hydrophobic phytonutrients using fenugreek galactomannan seed mucilage (FenuMat^®^) have emerged showing significant bioavailability and efficacy (Joseph et al., 2022a; Joseph et al., 2022b; Krishnakumar et al., 2022; Joseph et al., 2024; Abhilash et al., 2021). In this technology, hydrophobic and water-insoluble molecules and their nano structures such as liposomes, micelles, and emulsions are trapped within the fenugreek seed mucilage hydrogel scaffold. Upon dehydration, the hydrogel can be converted to water-soluble powder. Once ingested, it can rehydrate in the gastrointestinal tract to form a mucoadhesive hydrogel and further into a solution, leading to better absorption and bioavailability. Joseph et al. (2024) reported a full-spectrum bioavailability enhanced boswellia extract for both the volatile and non-volatile bioactives (hereinafter referred to as “F-BSE”) (Joseph et al., 2024). Abhilash et al. (2021) reported that curcuminoids can be co-formulated with boswellia extract as a single water-soluble powder with improved bioavailability for both curcuminoids and boswellic acids (hereinafter referred to as “C-BSE”) (Abhilash et al., 2021). Thus, the present randomized, placebo-controlled comparative study investigated the effects of F-BSE and C-BSE at a dosage of 400 mg/day on healthy volunteers suffering from moderate spondylitis issues characterized with pain and stiffness (n = 105).

## 2 Materials and methods

### 2.1 Study design

**FIGURE 1:**
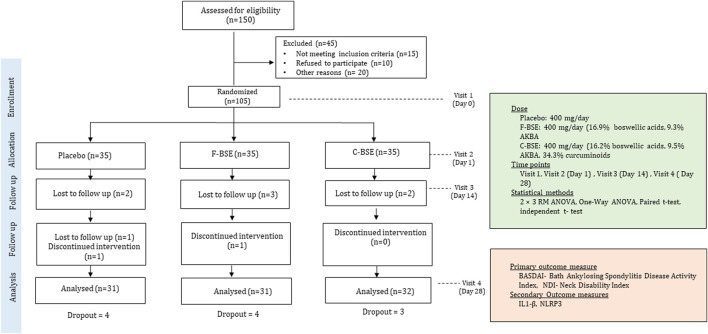
Diagram depicting research design.

This study was designed and carried out as a randomized, parallel group, double-blind, placebo-controlled trial, as depicted in the research design diagram (Figure 1). The protocol was approved by the registered Institutional Ethics Committee of Divakar’s Speciality Hospital, Bangalore, India, and was prospectively registered with the Clinical Trial Registry of India (CTRI/2021/12/038613 dated 14/12/2021). The sample size was determined through *a priori* power analysis using G*Power software, assuming an effect size (Cohen’s d = 0.2), a significance level (α) of 0.05, and a statistical power of 0.95. The analysis indicated that a total of 81 participants would be required, with 27 subjects per group in a three-arm design to account for statistical significance. To account for an anticipated 20% dropout rate, the minimum number of participants was adjusted to 33 participants per group. Accordingly, 35 participants were enrolled per group.

### 2.2 Participant recruitment and randomization

The potential participants were enrolled and assigned through the outpatient facility of Divakar’s Specialty Hospital and from the database of Leads Clinical Research and Bio Services Pvt. Ltd. Interested volunteers were screened on the basis of the inclusion/exclusion criteria, as detailed in Table 1. Written and informed consent was obtained prior to the commencement of the study. Recruited participants were randomized and allocated to any of the three groups (placebo, F-BSE, or C-BSE) in a 1:1:1 ratio using a computer-generated block randomization technique (www.randomization.com). An identification number was assigned to each participant on the basis of the order of enrolment in the study. The investigator was provided with randomization codes in separate envelopes by an independent statistician, which ensured the efficacy of the double-blinding procedure. Both subjective assessments (BASDAI and NDI) and objective biomarkers (IL-1β and NLRP3) were measured at baseline, day 14, and day 28 by trained staff blinded to treatment allocation. The blinding was maintained until all data were sealed for analysis to minimize bias.

**TABLE 1:** Inclusion and exclusion criteria.

Inclusion criteria
1. Male and female participants between 35 and 50 years of age.
2. Subjects with a known history of spondylitis difficulties.
3. Subject willing to adhere to their routine diet, physical activity, and general lifestyle throughout the study.
4. Subjects willing to give informed consent and comply with the guidelines of the study procedures.

Exclusion criteria
1. Subjects with any chronic, acute ailments or with any other diagnosed disorders or under medications and other therapies such as homoeopathy/Ayurveda.
2. Subjects having symptoms of viral infection, including COVID-19, HIV, or hepatitis B, or any other immuno-compromised state infection.
3. Subjects undergoing treatment for spondylitis.
4. Subjects allergic to herbal products or any component of the study product.
5. Subjects diagnosed with any other clinically significant medical condition, which in opinion of the investigator, may jeopardize the subject’s safety and preclude trial participation.
6. Subjects currently participating or having participated in another clinical trial during the last 3 months prior to the commencement of this study.

### 2.3 Interventions

Full-spectrum bioavailability enhanced *Boswellia* extract containing 16.9% of boswellic acids and 9.3% of acetyl-11-keto-β-boswellic acid (AKBA) (F-BSE) and its co-delivery form with curcumin having a composition of 16.2% total boswellic acids, 9.5% AKBA, and 34.3% of curcuminoids (C-BSE) formulated using the patented FenuMAT^®^ technology was provided by Akay Natural Ingredients, Cochin, India. The specification and analysis results of the test substance are provided in Supplementary Table S1.

Hard-shell gelatin capsules of placebo, F-BSE, and C-BSE (400 mg actives per capsule) were prepared based on good manufacturing practices (GMP) and were identically matched in terms of their color, size, and appearance. Additionally, the manufacturer provided a certificate of analysis and material safety data sheet, demonstrating the food-grade status. All the participants were advised to consume one capsule per day, with breakfast, for 28 days. Adherence to medication was confirmed by the counting pill strategy at each visit, and the effectiveness of blinding was concluded by asking the participants to predict the allocation.

All participants were allowed to use analgesic/anti-inflammatory gels as per their requirements. They were also allowed to use over-the-counter drug acetaminophen as a rescue medicine. However, participants were requested to note down the frequency of usage in the study diary.

### 2.4 Bath Ankylosing Spondylitis Disease Activity Index (BASDAI)

BASDAI is a validated questionnaire that is widely used worldwide to assess the pain, stiffness, and other difficulties associated with conditions such as spondylitis. It comprises six questions that assess the severity of pain at the neck/back/hip and other joints, morning stiffness, and fatigue level. Each question was rated on a 10-point Likert scale (Wiąk-Walerowicz and Wielosz, 2024; Cohen et al., 2006).

### 2.5 Neck Disability Index (NDI)

The NDI is a validated 10-item questionnaire for assessing the pain intensity during routine functions such as reading, driving, and work. Each item comprises six different assertions expressing progressive levels of pain/limitations in activities. Item scores range from 0 (no pain/limitation) to 5 (much pain/maximal limitation). The total score ranges from 0 to 50. The higher the score, greater the disability (Jorritsma et al., 2012).

### 2.6 Estimation of IL-1β and NLRP3 levels

Serum concentrations of IL-1β (catalog no: E-EL-H0149) and NLRP3 (E-EL-H2557) were analyzed with the respective ELISA kits following the manufacturer’s instruction, (Elabscience, Biotechnology Co., Limited. Bethesda, United States). The absorbance was read at 450 ± 2 nm employing a Varioskan™ LUX multimode microplate reader (Thermo Scientific™, Waltham, MA, United States).

### 2.7 Safety parameters

Biochemical tests were performed on an automated biochemical analyzer Cobas c501 (Roche–Hitachi, Manheim, Germany). Whole blood collected from the antecubital vein was used for the hematological analysis. To separate the serum, the clotted blood sample was centrifuged at 3,500 rpm for 10 min at 4°C, and the serum was stored at −80°C (Thomas et al., 2022). Liver function markers [aspartate aminotransferase (AST) and alanine aminotransferase (ALT)] were measured using standard kit methods (M/s Agappe Diagnostics Private Limited, Bangalore, India). The concentration of creatinine in the serum sample was analyzed by the method of Moss et al. (1975).

### 2.8 Statistical analysis

All collected data were transferred to a structured database and analyzed using SPSS version 28. Descriptive statistics were used to summarize the data: continuous variables were presented as mean ± standard deviations (SD) for normally distributed data and as medians with interquartile ranges (IQR) for non-normally distributed data; categorical variables were expressed as frequencies and percentages. The normality of distribution was assessed using the Shapiro–Wilk test (α = 0.05). Primary outcomes (BASDAI and NDI scores) were analyzed using a 2 × 3 repeated-measures ANOVA (group × time), followed by *post hoc* comparisons using pairwise and independent t-tests. Biomarker data were analyzed using one-way ANOVA for between-group comparisons and paired t-tests for within-group changes. Demographic and biochemical parameters were analyzed using pairwise or independent t-tests, as appropriate. All tests were two-sided, with a significance level set at p < 0.05. Adjustments for multiple comparisons were applied using Bonferroni correction where relevant. A quantitative comparison of rescue medication usage across groups was done using the chi-square test. Results were reported as mean changes from the baseline to the intermediate and final visits, along with associated p-values to indicate statistical significance.

## 3 Results

### 3.1 Study participants

**FIGURE 2:**
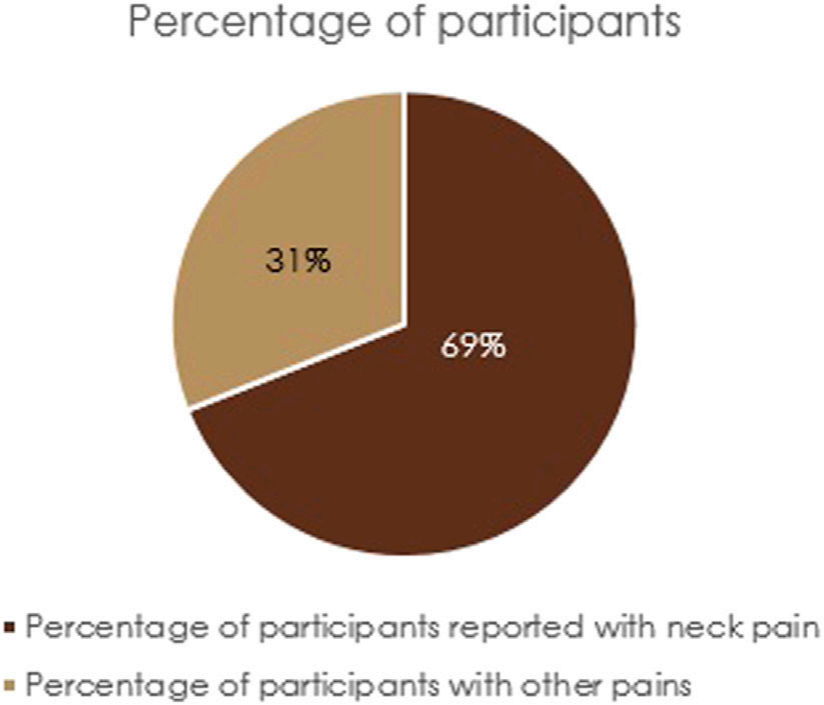
Percentage of participants reporting neck pain.

Detailed baseline demographics of the participants are provided in Table 2, which did not differ significantly between the placebo and intervention groups. All the participants were apparently healthy, except for mild-to-moderate spondylitis difficulties such as pain and stiffness in the neck, back, and hip, as evidenced from the baseline medical examination and BASDAI data. Of the 105 participants enrolled, 73 (69%) reported neck pain as their major issue, and they were randomized into three groups: 24 in the placebo, 25 in F-BSE, and 24 in C-BSE groups (Figure 2). Among the participants (n = 35/group) enrolled, only 93 participants completed the study. Four from the F-BSE, three from the C-BSE, and four from the placebo discontinued due to difficulty in complying with the inclusion criteria. The details of the research design are provided in Figure 1.

**TABLE 2:** Subject characteristics at baseline and on the 28th day.

Parameters	Groups	Day 0	Day 28
Age (years)	Placebo	28 ± 10.15	29 ± 9.89
	F-BSE	26 ± 10.26	26 ± 10.05
	C-BSE	28 ± 9.94	29 ± 9.88
Body weight (kg)	Placebo	69.6 ± 6.39	68.7 ± 4.94
	F-BSE	69.0 ± 9.07	69.2 ± 6.36
	C-BSE	67.4 ± 6.82	68.6 ± 6.23
BMI (kg/m^2^)	Placebo	25.4 ± 2.06	23.12 ± 2.42
	F-BSE	23.7 ± 2.53	23.95 ± 2.68
	C-BSE	24.9 ± 3.14	23.13 ± 2.19
Systolic blood pressure (mmHg)	Placebo	125.0 ± 7.95	122.0 ± 3.70
	F-BSE	124.0 ± 11.27	122.0 ± 6.76
	C-BSE	123.0 ± 10.88	121.0 ± 5.99
Diastolic blood pressure (mmHg)	Placebo	80.0 ± 7.26	84.0 ± 7.19
	F-BSE	81.0 ± 5.99	84.0 ± 5.64
	C-BSE	81.0 ± 7.84	83.0 ± 6.62
Pulse rate (/min)	Placebo	74.0 ± 9.66	74.0 ± 4.41
	F-BSE	79.0 ± 8.24	74.0 ± 2.09
	C-BSE	80.0 ± 9.10	73.0 ± 4.19

BMI, body mass index. Values are expressed as mean ± SD.

### 3.2 Influence of F-BSE and C-BSE on the BASDAI and NDI

#### 3.2.1 BASDAI

The total BASDAI score and the subscores (neck/hip/back pain, pain/swelling on other parts or joints, and morning stiffness) observed for the F-BSE and C-BSE groups in comparison with the placebo showed significant reduction with respect to both treatment and time by day 14 itself.

##### 3.2.1.1 Placebo vs. F-BSE

**FIGURE 3:**
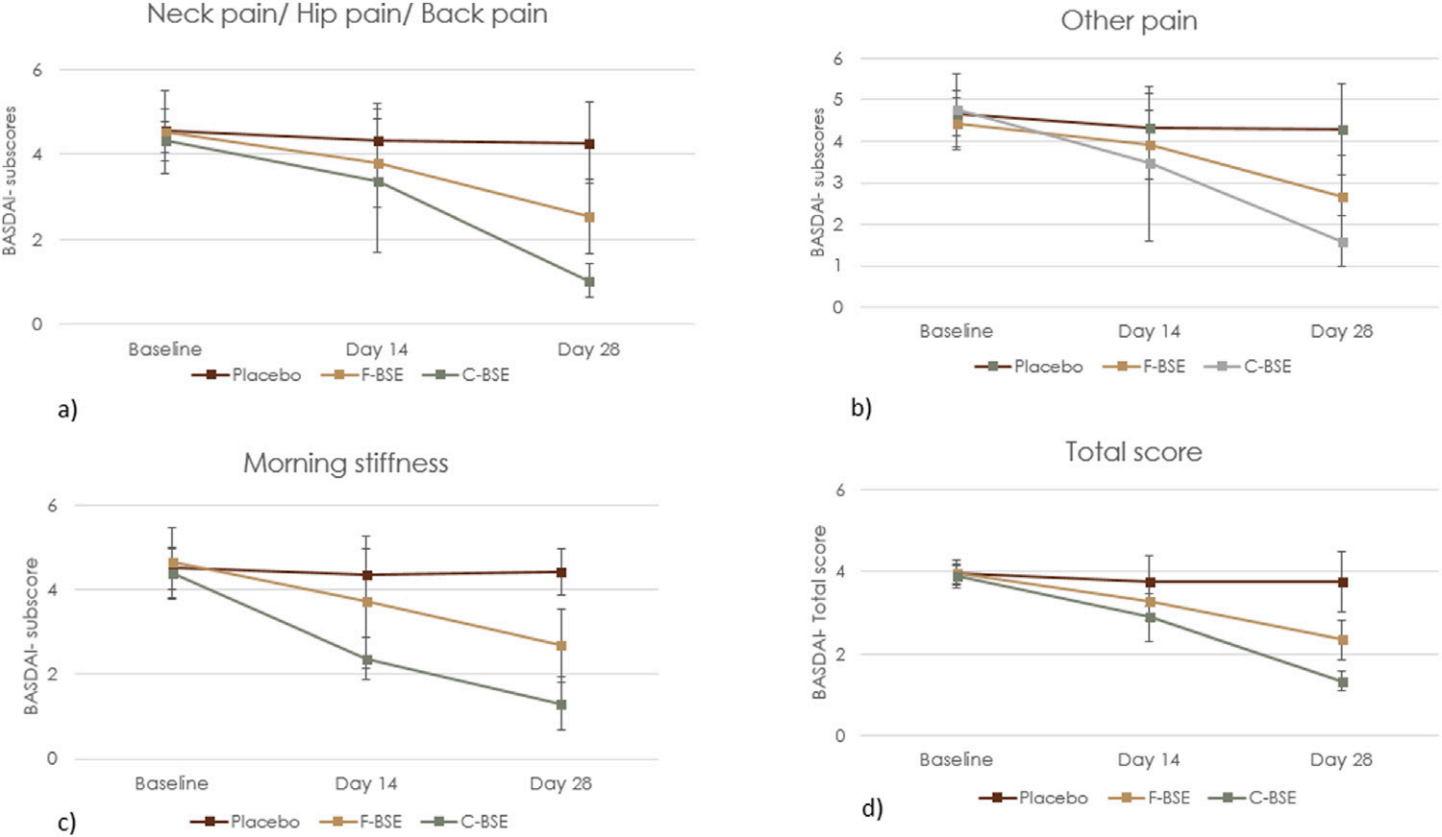
Mean change in the BASDAI questionnaire: **(a)** neck/hip/back pain; **(b)** other kinds of pain; **(c)** morning stiffness; **(d)** total BASDAI score. Values are expressed as mean ± SD; 2 × 3 repeated-measures ANOVA was performed to analyze statistical significance.

The total BASDAI score showed significant improvement in F-BSE compared to both placebo and baseline: treatment effect size: 0.850 [F (2, 62) = 169.58, 95% CI: 3.114, 3.282; p < 0.001], time effect size: 0.882 [F (2, 62) = 108.71, 95% CI: 2.942, 3.169; p < 0.001], and treatment × time effect size: 0.868 [F (2, 62) = 95.45, 95% CI: 2.165, 2.522; p < 0.001] (Table 3; Figure 3). The reduction in the subscores observed upon treatment with F-BSE is given as follows.

**TABLE 3:** Influence of F-BSE on BASDAI scores from a 2 × 3 RM ANOVA.

BASDAI data	Effects	Effect size	F-value	P-value
Neck, back, and hip pain	Treatment	0.796	116.72	<0.001
	Time	0.899	128.61	<0.001
	Treatment vs. time	0.865	93.10	<0.001
Other pain	Treatment	0.686	65.49	<0.001
	Time	0.855	85.82	<0.001
	Treatment vs. time	0.736	40.40	<0.001
Morning stiffness	Treatment	0.534	457.14	<0.001
	Time	0.938	162.03	<0.001
	Treatment vs. time	0.918	106.53	<0.001
Total BASDAI	Treatment	0.938	457.24	<0.001
	Time	0.968	442.76	<0.001
	Treatment vs. time	0.970	470.29	<0.001

Repeated-measure ANOVA, with p-values, F-values, and effect size for subjective reports. P < 0.05 is considered statistically significant.

The pain score corresponding to the neck/back/hip showed a treatment effect size of 0.497 [F (2, 62) = 29.64, 95% CI: 3.382, 3.866; p < 0.001] and time effect size of 0.699 [F (2, 62) = 33.71, 95% CI: 3.169, 3.637; p < 0.001]. The treatment × time effect size was 0.475 [F (2, 62) = 13.11, 95% CI: 2.223. 2.874; p < 0.001], indicating the positive response of F-BSE (Figure 3). Pairwise and independent t-tests further confirmed the effect of F-BSE compared to the baseline and placebo. The percentage reduction with respect to the baseline was found to increase from 15.72% on day 14 (p < 0.001) to 43.58% by day 28 (p < 0.001) and by 40.15% compared to that of the placebo (p < 0.001) [Supplementary Table S2A, B].

The overall levels of pain/swelling, other than those in the neck/back/hip, also reduced with respect to treatment [effect size: 0.633, F (2, 62) = 51.84, 95% CI: 3.524, 3.788; p < 0.001], time [effect size: 0.530, F (2, 62) = 16.33, 95% CI: 3.147, 3.789; p < 0.001], and treatment × time [effect size: 0.487, F (2, 62) = 13.75, 95% CI: 2.72, 3.019; p < 0.001] (Figure 3). The relative decrease from baseline was 11.68% (day 14) to 40.14% (p < 0.001) by day 28 and 38.34% (p < 0.001) compared to that of placebo [Supplementary Table S2A,B].

The effect of intervention on morning stiffness was also significant with respect to time [effect size: 0.675, F (2, 62) = 30.10, 95% CI: 3.335, 3.762; p < 0.001] and treatment [effect size: 0.534, F (2, 62) = 34.37, 95% CI: 3.436, 3.919; p < 0.001] (Figure 3). Both inter- and intra-group comparisons further demonstrated significant improvement by day 14 [Supplementary Table S2A,B].

##### 3.2.1.2 Placebo vs. C-BSE

C-BSE supplementation significantly improved the BASDAI total score, thus indicating its efficacy over placebo, which further significantly progressed through day 28 with a treatment effect size of 0.938 [F (2, 63) = 457.24, 95% CI: 2.614, 2.811; p < 0.001] and the time effect size of 0.968 [F (2, 63) = 442.76, 95% CI: 2.485, 2.639; p < 0.001]. The treatment × time interaction was also significant, with an effect size of 0.970 [F (2, 63) = 470.29, 95% CI: 1.264, 1.449; p < 0.001] (Figure 3; Table 4).

**TABLE 4:** Influence of C-BSE on BASDAI scores from a 2 × 3 RM ANOVA.

BASDAI data	Effects	Effect size	F-value	P-value
Neck, back, and hip pain	Treatment	0.497	29.64	<0.001
	Time	0.699	33.71	<0.001
	Treatment vs. time	0.475	13.11	<0.001
Other pain	Treatment	0.633	51.84	<0.001
	Time	0.530	16.33	<0.001
	Treatment vs. time	0.487	13.75	<0.001
Morning stiffness	Treatment	0.534	34.37	<0.001
	Time	0.675	30.10	<0.001
	Treatment vs. time	0.687	31.89	<0.001
Total BASDAI	Treatment	0.850	169.58	<0.001
	Time	0.882	108.71	<0.001
	Treatment vs. time	0.868	95.45	<0.001

Repeated-measure ANOVA, with p-values, F-values, and effect size for subjective reports. P < 0.05 is considered statistically significant.

The following relative decreases were found in the subscores: pain at the neck/hip/back [treatment effect size: 0.796 (F (2, 63) = 116.72, 95% CI: 2.702,3.147; p < 0.001), time effect size: 0.899 (F (2, 63) = 128.61, 95% CI: 2.461, 2.830; p < 0.001), and the treatment × time effect size: 0.865 (F (2, 63) = 93.10, 95% CI: 0.883, 1.182; p < 0.001)]. For pain/swelling in other body parts, the observed treatment effect size was 0.686 [F (2, 63) = 65.49, 95% CI: 2.952, 3.500; p < 0.001], time effect size was 0.855 [F (2, 63) = 85.82, 95% CI: 2.743, 3.160; p < 0.001], and the treatment × time effect size was 0.736 [F (2, 63) = 40.40, 95% CI: 1.387, 1.839; p < 0.001]. Similar improvement was observed for morning stiffness with a treatment effect size: 0.938 [F (2, 63) = 457.14, 95% CI: 2.584, 2.835; p < 0.001], time effect size: 0.918 [F (2, 63) = 162.03, 95% CI: 2.729, 3.013; p < 0.001], and the treatment × time effect size: 0.880 [F (2, 63) = 106.53, 95% CI: 1.083, 1.562; p < 0.001].

Furthermore, pairwise comparison of treatment (placebo × C-BSE) showed a significant effect of C-BSE (p < 0.001) in reducing pain in the neck/hip/back, other pain/swelling, and morning stiffness by day 14 itself, based on subscore analyses comparing baseline data with those of days 14 and 28. The percentage changes observed on supplementation with C-BSE were as follows: neck/hip/back pain (day 14: 21.73%; p = 0.004; day 28: 76.09%; p < 0.001), pain/swelling on other parts or joints (day 14: 26.99%; p < 0.001; day 28: 66.46%; p < 0.001), morning stiffness (day 14: 45.71%; p < 0.001; day 28: 70.01%; p < 0.001), and total score (day 14: 25.52%; p < 0.001; day 28: 65.46%; p < 0.001). The between-group effect of placebo vs. C-BSE also exhibited a significant decrease in the subscores [Supplementary Table S2A, B].

Compared to F-BSE, C-BSE showed improved effects, and the observed improvements for stiffness and total BASDAI were significant on day 14 (p < 0.001). Both F-BSE and C-BSE groups showed significant improvements over the 28-day supplementation. By day 28, C-BSE demonstrated decreased subscores and the total BASDAI score (p < 0.001) [Supplementary Table S2B].

#### 3.2.2 NDI

##### 3.2.2.1 Placebo vs. F-BSE

**FIGURE 4:**
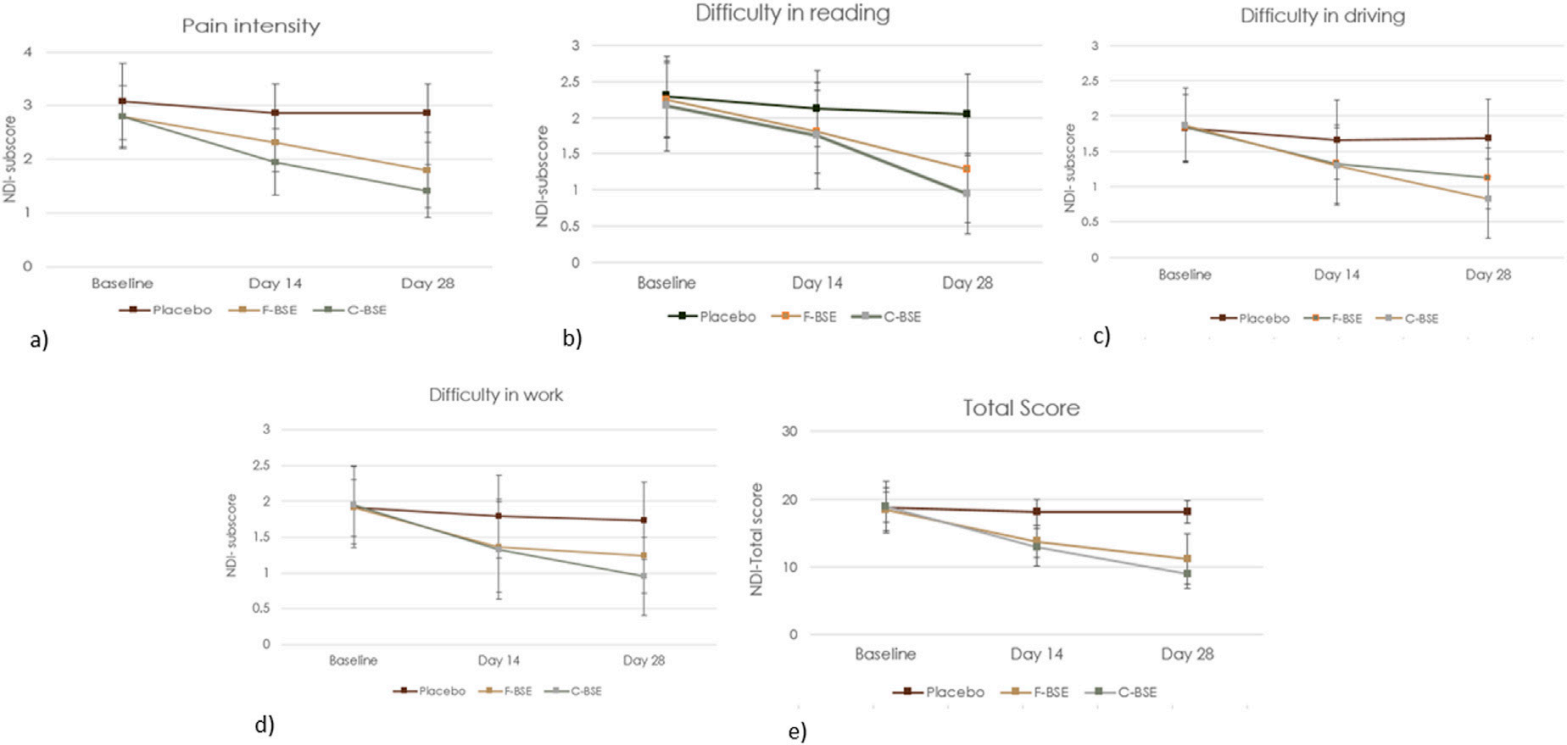
Mean change in the NDI questionnaire: **(a)** pain intensity; **(b)** difficulty in reading, **(c)** difficulty in driving; **(d)** difficulty in work; **(e)** total NDI score. Values are expressed as mean ± SD; 2 × 3 repeated-measures ANOVA was performed to analyze statistical significance.

The observed reductions in various subscores are summarized as follows. Pain intensity showed a significant treatment effect size of 0.575 [F (2, 49) = 29.76, 95% CI: 2.103, 2.447; p < 0.001] and time effect size of 0.685 [F (2, 49) = 22.85, 95% CI: 2.125, 2.440; p < 0.001]. Treatment × time interaction effect size was 0.518 [F (2, 49) = 11.29, 95% CI: 1.421, 1.970; p < 0.001]. Significant improvements were also observed with respect to difficulties in reading and driving (Figure 4; Table 5).

**TABLE 5:** Influence of F-BSE on NDI scores from a 2 × 3 RM ANOVA.

NDI data	Effects	Effect size	F-value	P-value
Pain intensity	Treatment	0.575	29.76	<0.001
	Time	0.685	22.85	<0.001
	Treatment vs. time	0.518	11.29	<0.001
Difficulty in reading	Treatment	0.306	9.70	0.005
	Time	0.568	13.82	<0.001
	Treatment vs. time	0.443	8.36	0.002
Difficulty in driving	Treatment	0.585	30.95	<0.001
	Time	0.464	9.07	0.001
	Treatment vs. time	0.585	14.78	<0.001
Difficulty in work	Treatment	0.263	7.83	0.010
	Time	0.466	9.17	0.001
	Treatment vs. time	0.229	3.11	0.065
Total NDI	Treatment	0.665	43.71	<0.001
	Time	0.799	41.84	<0.001
	Treatment vs. time	0.789	39.27	<0.001

Repeated-measure ANOVA, with p-values, F-values, and effect size for subjective reports. P < 0.05 is considered statistically significant.

Pairwise comparison of placebo and F-BSE treatments confirmed the significant effects of F-BSE by day 14, as evident from the reduction in pain intensity; difficulty in reading, driving, and work; and total score on day 14 and day 28. The percentage changes observed for F-BSE were as follows: pain intensity (day 14: 19.16%; p < 0.001; day 28: 37.06%; p < 0.001), difficulty in reading (day 14: 15.09%; p = 0.047; day 28: 37.25%; p < 0.001), difficulty in driving (day 14: 20.48%; p = 0.006; day 28: 33.73%; p < 0.001), difficulty in work (day 14: 24.02%; p = 0.018; day 28: 28.32%; p = 0.002), and total score (day 14: 23.73%; p < 0.001; day 28: 38.67%; p < 0.001). Intra-group comparisons also showed the significant effects of F-BSE [Supplementary Table S3A, B].

##### 3.2.2.2 Placebo vs. C-BSE

The influence of C-BSE supplementation on various discomforts is presented in Figure 4 and Table 6, which includes the effect sizes and significance levels for treatment, time, and the treatment-by-time interaction. Both the total NDI score and the subscores showed significant improvements (p < 0.001).

**TABLE 6:** Influence of C-BSE on NDI scores from a 2 × 3 RM ANOVA.

NDI data	Effects	Effect size	F-value	P-value
Pain intensity	Treatment	0.666	43.82	<0.001
	Time	0.742	30.15	<0.001
	Treatment vs. time	0.618	17.01	<0.001
Difficulty in reading	Treatment	0.546	26.46	<0.001
	Time	0.631	17.97	<0.001
	Treatment vs. time	0.533	12.00	<0.001
Difficulty in driving	Treatment	0.461	18.78	<0.001
	Time	0.779	37.10	<0.001
	Treatment vs. time	0.394	6.81	0.005
Difficulty in work	Treatment	0.342	11.42	0.003
	Time	0.552	12.93	<0.001
	Treatment vs. time	0.603	15.97	<0.001
Total NDI	Treatment	0.752	66.56	<0.001
	Time	0.858	63.49	<0.001
	Treatment vs. time	0.825	49.61	<0.001

Repeated-measure ANOVA, with p-values, F-values, and effect size for subjective reports. P < 0.05 is considered statistically significant.

Further pairwise analysis also revealed the significant effects of C-BSE treatment (p < 0.001), especially on pain intensity and NDI subscores, compared to that of placebo, by days 14 and 28 of the study period [Supplementary Table S2A, B].

The relative effects of C-BSE and F-BSE were also analyzed. Although both treatments significantly reduced pain intensity and difficulties with reading, driving, and work by day 14, C-BSE showed greater improvement than F-BSE on day 28, especially in pain intensity (p < 0.05) [Supplementary Table S3B].

### 3.3 Influence of F-BSE and C-BSE on IL-1β and NLRP3 concentrations

**FIGURE 5:**
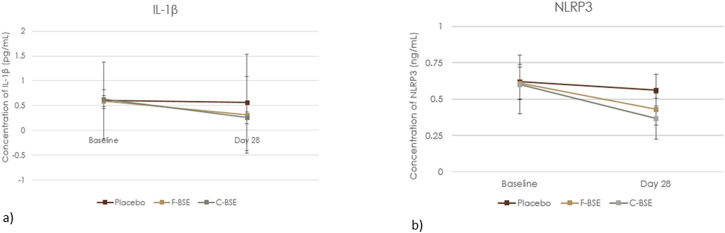
Mean change in **(a)** IL-1β and **(b)** NLRP3. Values are expressed as Mean ±SD, 2 × 3 repeated measures ANOVA was analysed to perform statistical significance.

Statistical analysis showed a significant decrease in IL-1β and NLRP3 concentrations following supplementation with F-BSE and C-BSE compared to both those of baseline and placebo (Figure 5). However, placebo treatment did not produce a significant change. The relative change observed upon treatment and its comparison is detailed in Supplementary Table S3. At the end of the study, the percentage change upon within-group analysis was 46.62%, p < 0.001 (IL-1β) and 29.62%, p < 0.001 (NLRP3) for IL-1β and NLRP3, respectively, for F-BSE and 59.33%, p < 0.001 and 39.27%, p < 0.001 for IL-1β and NLRP3, respectively, for C-BSE.

Compared to the placebo, C-BSE treatment reduced IL-1β concentration by 54.63% (p < 0.001) and that of NLRP3 by 35.05% (p < 0.001). F-BSE treatment also exhibited significant reductions of 43.77% for IL-1β and 23.49% for NLRP3 (p < 0.001) Supplementary Table S4.

### 3.4 Rescue medicine usage

Anti-inflammatory/pain-relieving topical gels containing diclofenac and capsaicin and over-the counter drugs were the primary rescue medicines used in the study. At baseline, 77% of participants from the placebo group, 83% from F-BSE group, and 86% from C-BSE group reported using gels 2–4 times daily for 5–7 days/week. The percentage of oral medication users was 43%, 51%, and 57% in the placebo, F-BSE, and C-BSE groups, respectively. Upon treatment for 14 days, the frequency of gel usage was reduced to 1–3 times per day, and it further reduced to 0–2 times by day 28. Notably, 21% of the participants in the F-BSE group and 27% in the C-BSE group discontinued gel usage by day 14, increasing to 29% and 40%, respectively, by day 28. In contrast, gel usage in the placebo group decreased from 23% at the baseline to 20% on day 14 and 17% on day 28.

**FIGURE 6:**
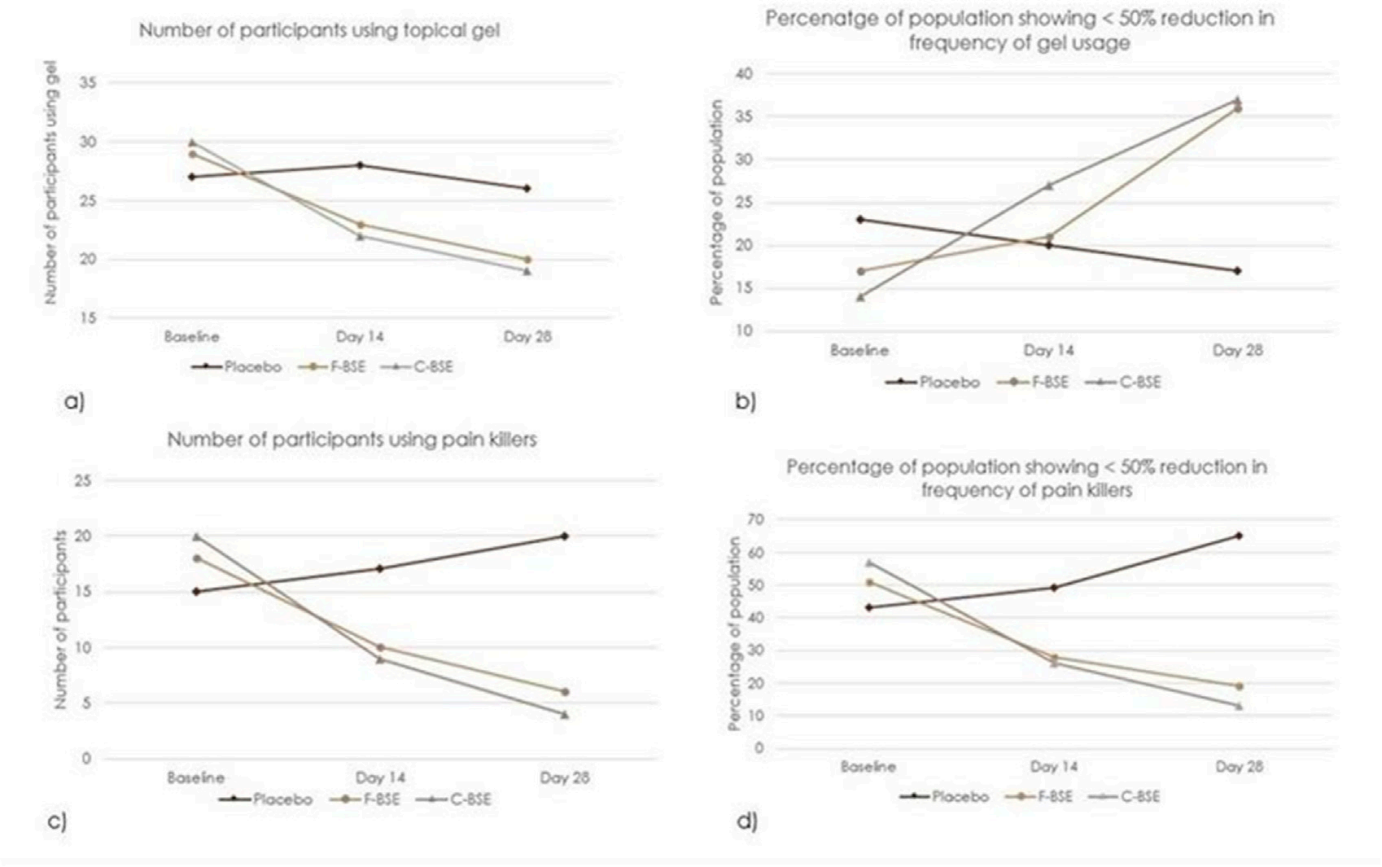
Percentage and frequency of rescue medicine usage in placebo, F-BSE, and C-BSE groups over time. **(a)** Number of participants using pain killers. A chi-square test of independence was performed to evaluate the significance between groups on topical gel usage. The result was significant, X2 (4, N = 94) = 1.47, p < 0.05. **(b)** Percentage of population showing <50% reduction in frequency of gel usage. **(c)** Number of participants using pain killers. A chi-square test of independence was performed to evaluate the significance between groups on topical gel usage. The result was significant, X2 (4, N = 94) = 12. 17. p < 0.01. **(d)** Percentage of population showing <50% reduction in frequency of pain killers.

Oral medicine usage also decreased significantly upon treatment with F-BSE and C-BSE. While the percentage of oral medicine users in the placebo group increased from 43% to 56% by day 28, the F-BSE and C-BSE groups showed a decrease in both the number of users and the frequency of usage. The observed reduction in the percentage of participants on treatment with F-BSE was 28% on day 14 and 20% on day 56. C-BSE treatment showed a better effect, with 26% reduction on day 14 and 11% by day 28 (Figure 6).

### 3.5 Safety and tolerance of F-BSE and C-BSE

Both F-BSE and C-BSE were well-tolerated and did not produce any major side effects or adverse events, except for a couple of participants from each group who reported dryness of throat, abdominal fullness, and appetite reduction in the initial days for 1 week. The analysis of biochemical safety parameters showed no significant difference (p > 0.05), neither from the baseline nor from the normal range, at the end of the study (Table 7).

**TABLE 7:** Influence of placebo, F-BSE, and C-BSE on biochemical safety markers.

Parameters	Groups	Day 0	Day 28
AST (U/L)	Placebo	24.43 ± 6.93	30.85 ± 9.65
	F-BSE	25.87 ± 7.25	31.02 ± 9.29
	C-BSE	33.93 ± 14.11	32.11 ± 9.07
ALT (U/L)	Placebo	26.86 ± 11.69	31.92 ± 14.19
	F-BSE	23.07 ± 13.0	30.79 ± 7.25
	C-BSE	35 ± 20.88	30.73 ± 11.12
Serum creatinine (mg/dL)	Placebo	0.88 ± 0.14	0.86 ± 0.11
	F-BSE	0.86 ± 0.14	0.84 ± 0.15
	C-BSE	0.76 ± 0.13	0.85 ± 0.13

ALT, alanine aminotransferase; AST, aspartate aminotransferase. Values are expressed as mean ± SD. The values bearing a superscript “*” significantly differ at p < 0.05 from its baseline or comparator.

## 4 Discussion

In the present randomized, parallel group, double-blind, placebo-controlled study, supplementation with F-BSE and C-BSE was found to significantly improve the pain severity and joint stiffness among subjects characterized with mild-to-moderate spondylitis issues. The average baseline BASDAI scores of the selected participants were 3.96 ± 0.24, 3.97 ± 0.30, and 3.88 ± 0.26, respectively, for the placebo-, F-BSE-, and C-BSE-treated groups, indicating moderate pain/swelling/stiffness issues due to inflammation and limited mobility. According to the Spondyloarthritis International Society criteria, BASDAI score ≥4 corresponds to severe conditions of pain and stiffness, while a score <4 indicates low disease intensity (Wiąk-Walerowicz and Wielosz, 2024). A score <2 was considered indicating remission (Cohen et al., 2006). The BASDAI questionnaire has been validated as a good self-reported assessment tool on various aspects of disease severity (Madsen, 2018).

Both F-BSE- and C-BSE-treatments resulted in a notable reduction in the total BASDAI score and the subscores related to neck/back/hip pain, pain/swelling on other joints, and morning stiffness compared to those of both the baseline and placebo. Further pairwise and independent analyses revealed the distinct effects of the treatments (placebo, F-BSE, and C-BSE) on days 7, 14, and 28. It can be seen that the improvement was evident from day 7 onward in both the F-BSE and C-BSE groups, with a marked reduction in pain and stiffness by day 14, which continue to progress through day 28. However, C-BSE administration demonstrated more significant effects than F-BSE, especially on pain at the neck/hip (59.53%; p < 0.001) and stiffness (50.98%; p < 0.001).

Among the enrolled participants, approximately 70% mentioned neck pain as their major concern. So, we employed NDI to assess the disability and mobility limitations associated with neck pain (Aljinović et al., 2023). The significant decrease observed in NDI scores, compared to baseline, indicated the primary efficacy of F-BSE and C-BSE. The extent of reduction in the treatment groups related to placebo further demonstrates their clinical relevance. Comparative analysis using t-tests provided additional evidence for the relative efficacy of F-BSE and C-BSE in relation to placebo and the superior effect of C-BSE in comparison with F-BSE (F-BSE vs. C-BSE) during a span of 14 and 28 days.

The therapeutic efficacy of F-BSE and C-BSE may be attributed to the anti-inflammatory and anti-nociceptive effects of boswellia extract and curcumin (Sethi et al., 2022). The relatively better effect of C-BSE, particularly by day 28, may be explained by the synergistic action of curcumin and boswellic acids when administered in a bioavailable manner. This is supported by our analysis of NLRP3 inflammasome and IL-1β levels. The NLRP3 inflammasome triggers the activation of caspase-1, promoting the release of proinflammatory mediators and contributing to pain perception through immune system interactions, while IL-1β serves as a key inflammatory mediator and has a significant role in both the induction and maintenance of pain during chronic inflammatory conditions (Li et al., 2020; Ren and Torres, 2009). Supplementation of both F-BSE and C-BSE significantly reduced the levels of NLRP3 and IL-1β.

Boswellia oleo-gum resin and turmeric rhizomes have been widely used in Ayurveda for the treatment of inflammatory diseases and pain management. Their key bioactives include both non-volatile (pentacyclic triterpenoid boswellic acids and polyphenolic curcuminoids) and volatile (terpenes and terpenoids) constituents. Boswellic acids act on multiple molecular targets involved in inflammation and pain, such as 5-lipoxygenase (5-LOX), cyclooxygenase-1 (COX-1), mitogen-activated protein kinase (MAPK), microsomal prostaglandin-E synthase (mPGES)-1, nuclear transcription factor-κB (NF-κB), pro-inflammatory cytokines such as tumor necrosis factor-α (TNFα), and proinflammatory interleukins and are involved in inhibition of histamines and nitric oxide (Joseph et al., 2024; Sharma and Jana, 2020). They are also known to inhibit TLR4 and IL1R signaling and downregulate the NLRP3 inflammasome pathway (Franco-Trepat et al., 2023; Mobasheri and Henrotin, 2018). Among the boswellic acids, AKBA is the most potent inhibitor of 5-lipoxygenase and leukotriene-mediated inflammatory pathways (Yu et al., 2020; Majeed et al., 2021). F-BSE is a full-spectrum boswellia formulation containing 10% AKBA, with enhanced bioavailability for both the volatile and non-volatile bioactives (Joseph et al., 2024).

The antioxidant, anti-inflammatory, analgesic, and anticancer properties of curcumin have been well investigated. It exhibits a pleiotropic mechanism of action involving transcription factors, such as NF-kB, activated protein-1(AP-1), signal transducer and activator of transcription (STAT) proteins, peroxisome proliferator-activated receptor-γ (PPAR-γ), and β-catenin (Fadus et al., 2017). Additionally, curcumin directly inhibits NLRP3 inflammasome assembly or suppresses its activation through the inhibition of the NF-κB pathway (Peng et al., 2021). However, the clinical efficacy of curcumin has been shown to be limited by the poor oral bioavailability of the anti-inflammatory free (unconjugated) curcuminoids (Kumar et al., 2016).

Previously, research has demonstrated the enhanced bioavailability of a food-grade formulation of curcumin (CGM; registered as CurQfen^®^) and a co-delivery system combining curcumin and boswellia extract using fenugreek galactomannan soluble dietary fiber (mucilage) as a non-covalent water-dispersible molecular complex (patented FENUMAT^®^ technology) (Abhilash et al., 2021; Kumar et al., 2016). The bioavailability enhanced co-delivery system of curcumin and boswellia extract (C-BSE) is used in the current study. The synergistic effects of curcumin and boswellia extract in alleviating pain and inflammation in osteoarthritic participants have already been reported (Sethi et al., 2022). A previous study reported the effects of a curcumin and Boswellia combination at 500 mg × 3/day (Curamin^®^; 350 mg curcuminoids and 150 mg Boswellia extract) during a 12-week study period (Haroyan et al., 2018). The improved efficacy of C-BSE at a lower dose of 400 mg/day over 14 days of treatment in reducing the pain and stiffness may be attributed to the improved bioavailability, which delivers a full spectrum of bioactives in turmeric and boswellia gum oleo-resin.

## 5 Conclusion

Herbal extracts with anti-inflammatory and analgesic properties are widely utilized for the management of musculoskeletal pain/stiffness issues. Curcumin and boswellic acids have demonstrated potent anti-inflammatory effects and have been reported to alleviate pain and stiffness associated with musculoskeletal disorders such as osteoarthritis. However, long-term supplementation (such as 12 weeks) and high dosage (1–2 g/day) are usually suggested due to their poor oral bioavailability. The present randomized, double-blind, placebo-controlled, three-arm, parallel group comparative study evaluated the relative efficacy of bioavailability enhanced formulations of the full-spectrum extract of *Boswellia serrata* oleo gum-resin (F-BSE) and its co-delivery system with curcumin (C-BSE) in alleviating the pain/stiffness issues associated with mild-to-moderate spondylitis. A relatively low dosage of 400 mg/day once daily was used, and the symptoms were monitored for a short duration of 14–28 days. Both F-BSE and C-BSE significantly reduced total and subscores of BASDAI, indicating alleviation of pain and stiffness by day 14. Further assessment using the NDI revealed a reduction in neck pain and an improvement in mobility-related impairments among participants with high baseline neck pain scores. The observed improvement continued to progress to day 28. C-BSE demonstrated a more pronounced effect than F-BSE. Additionally, both compounds suppressed the levels of the pro-inflammatory cytokines IL-1β and NLRP3 inflammasome, suggesting a reduction in pain through inhibiting the synthesis of inflammatory mediators and modulating pain pathways in the peripheral and central nervous systems. However, future research with extended duration of treatment and a broader evaluation of biochemical markers would be helpful to substantiate the synergistic anti-inflammatory and anti-nociceptive effects of these formulations as a complementary approach in the management of spondylitis and spondylosis. Although F-BSE and C-BSE showed significant effects on inflammatory markers and clinical symptoms, the 28-day intervention period and small sample size (n = 105) limit the broader applicability and long-term interpretation of the findings. Further research with larger cohorts and extended follow-up is recommended. Another limitation of the study is the inability to perform the analyses according to the intention-to-treat criteria, which would have provided additional insight by preserving the benefits of randomization and accounting for all enrolled participants. The exclusion of dropouts may limit the generalizability of the findings to real-world settings, where adherence may vary. Future studies are also recommended to include X-ray or MRI techniques for screening, which remains as a limitation of the present study.

## Data Availability

The original contributions presented in the study are included in the article/Supplementary Material further enquiries can be directed to the corresponding author.
