# Supplementary material for: A full-spectrum Boswellia serrata extract with enhanced bioavailability, and its co-delivered system with curcumin alleviate pain and stiffness associated with moderate spondylitis: a randomized double-blind, placebo-controlled, 3-arm study
Source: Front Pharmacol. 2025 Jul 1;16:1577429. doi: 10.3389/fphar.2025.1577429 (PMC12260406; doi:10.3389/fphar.2025.1577429)
Supplement: Supplementary file 1 [file Supplementaryfile1.docx]

Supplementary Table

The specification and analysis results of the test substances used in the study and the influence of F-BSE and C-BSE on BASDAI, NDI Questionnaire and biomarkers

**Supplementary Table S1**: The specification and analysis results of the test substances used in the present study

| **Parameters** | **Test method** | **Specification** | **F-BSE** | **C-BSE** |
| --- | --- | --- | --- | --- |
| *Physical characteristics* | | | | |
| Colour and Appearance |  | Powder | Creamish brown powder | Yellow powder |
| Particle size (g/mL) | USP 29 <786> | NLT 98% thru. 20 mesh | 99.2% | 99.5% |
| Bulk density | USP 29 <786> | 0.3 - 0.6 | 0.45 | 0.58 |
| *Chemical characteristics* | | | | |
| Total Boswellic acids content | HPLC | 15 -18% | 16.9% | 16.2% |
| Acetyl-11-keto-β-boswellic acid (AKBA) | HPLC | 8-10% | 9.3% | 9.5% |
| Total curcumin content | HPLC | 34-36% | NIL | 34.3% |
| Moisture content | USP 29 < 921> KF | NMT 6% | 3.88% | 2.1% |
| *Residual solvents** | | | | |
| Ethanol | As per USP | USP 30 <467> | 23 ppm | 68 ppm |
| *Heavy Metals (ppm)#* | | | | |
| Lead | AOAC, 2015.01 | < 2 | 0.15 | 0.19 |
| Mercury | AOAC, 2015.01 | < 1 | Not detected | Not detected |
| Cadmium | AOAC, 2015.01 | < 1 | Not detected | Not detected |
| Arsenic | AOAC, 2015.01 | < 2 | Not detected | Not detected |
| *Mycotoxins (ppb)$* | | | | |
| B1+B2 & G1+G2 | HPLC ASTA Method 24.2 | < 4.0 | conforms | conforms |
| Aflatoxin B1 | HPLC ASTA Method 24.2 | < 2.0 | conforms | conforms |
| Ochratoxin | Vicam method, AOAC | < 15 | conforms | conforms |
| *Microbiology*** | | | | |
| Total Plate count | FDA BAM Ch 3, 2001 | < 10,000 cfu/g | 500 cfu/g | 200 cfu/g |
| Yeast & mold | FDA BAM Ch 18, 2001 | < 100 cfu/g | 20 cfu/g | 30 cfu/g |
| E. Coli | FDA BAM Ch 4, 2020 | Absent/g | Absent/g | Absent/g |
| Salmonella | FDA BAM Ch 5, 2011 | Absent/25g | Absent/25g | Absent/25g |
| Coliforms | FDA BAM Ch 4, 2020 | Absent | Absent | Absent |
| *Lot No. and Manufacturing date* | - |  | 18/06/2021  & 9520001 | 17/10/2020  & 3800154 |

*****Residual solvents were analyzed by approved methods of United states pharmacopeia; USP <467>; ***^#^***Heavy metals were analyzed by Association of Official Analytical Chemists (AOAC) official method 2015.01; ***^$^***Mycotoxins B1, B2, G1, G2 and Ochratoxins were analyzed by: American Spice Trade Association (ASTA) method; Chapter 24.2, Jan 1997; and AOAC approved methods; AOAC 991.44-1996(2002); ********Microbiology parameters were analyzed by FDA BAM methods (Food and Drug Administration – Bacteriological Analytical Manual).

**Supplementary Table S2(a)**: Mean ± SD and P value of BASDAI questionnaire upon intragroup comparison

| Parameters | Groups | Mean ± SD | | | | Intragroup comparison | | | |
| --- | --- | --- | --- | --- | --- | --- | --- | --- | --- |
|  |  |  |  |  |  | Day 1 vs Day 14 | | Day 1 vs Day 28 | |
|  |  | Day1 | Day14 | Day28 | % difference | | P value | % difference | P value |
| Neck/hip/back pain | Placebo | 4.54 ± 0.50 | 4.32 ± 0.87 | 4.25 ± 0.96 | | 4.97 | 0.198 | 6.38 | 0.163 |
|  | F-BSE | 4.51 ± 0.96 | 3.80 ± 1.04 | 2.54 ± 0.88 | | 15.72 | 0.002 | 43.58 | < 0.001 |
|  | C-BSE | 4.31 ± 0.47 | 3.37 ± 1.68 | 1.03 ± 0.40 | | 21.73 | 0.004 | 76.09 | < 0.001 |
| Other pain | Placebo | 4.67 ± 0.54 | 4.32 ± 0.83 | 4.29 ± 1.10 | | 7.59 | 0.086 | 8.27 | 0.123 |
|  | F-BSE | 4.41 ± 0.62 | 3.90 ± 0.83 | 2.64 ± 1.01 | | 11.68 | 0.016 | 40.14 | < 0.001 |
|  | C-BSE | 4.75 ± 0.87 | 3.46 ± 1.86 | 1.59 ± 0.61 | | 26.99 | < 0.001 | 66.46 | < 0.001 |
| Morning stiffness | Placebo | 4.51 ± 0.50 | 4.35 ± 0.60 | 4.41 ± 0.56 | | 3.67 | 0.305 | 2.14 | 0.500 |
|  | F-BSE | 4.64 ± 0.83 | 3.70 ± 1.55 | 2.67 ± 0.87 | | 20.15 | 0.003 | 42.37 | < 0.001 |
|  | C-BSE | 4.37 ± 0.60 | 2.37 ± 0.49 | 1.31 ± 0.64 | | 45.71 | < 0.001 | 70.01 | < 0.001 |
| Total score | Placebo | 3.96 ± 0.24 | 3.77 ± 0.62 | 3.76 ± 0.73 | | 4.72 | 0.103 | 5.05 | 0.151 |
|  | F-BSE | 3.97 ± 0.30 | 3.27 ± 0.46 | 2.34 ± 0.48 | | 17.59 | < 0.001 | 41.04 | < 0.001 |
|  | C-BSE | 3.88 ± 0.26 | 2.89 ± 0.59 | 1.34 ± 0.25 | | 25.52 | < 0.001 | 65.46 | < 0.001 |

BASDAI-Bath Ankylosing Spondylitis Disease Activity Index. Values are expressed as mean ± SD. P < 0.05 is considered as statistically significant.

**Supplementary Table S2(b)**: Percentage change and P value of BASDAI questionnaire upon intergroup comparison

| Parameters | Placebo vs F-BSE | | | | Placebo vs C-BSE | | | | F-BSE vs C-BSE | |
| --- | --- | --- | --- | --- | --- | --- | --- | --- | --- | --- |
|  | Day14 | | Day 28 | | Day14 | | Day 28 | | Day14 | Day 28 |
|  | % difference | P value | % difference | P value | % difference | P value | % difference | P value | P value | P value |
| Neck/hip/back pain | 11.93 | 0.039 | 40.15 | < 0.001 | 21.91 | 0.007 | 75.78 | < 0.001 | 0.228 | < 0.001 |
| Other pain | 9.69 | 0.052 | 38.34 | < 0.001 | 19.75 | 0.023 | 62.86 | < 0.001 | 0.240 | < 0.001 |
| Morning stiffness | 14.81 | 0.035 | 39.42 | < 0.001 | 45.45 | < 0.001 | 70.31 | < 0.001 | < 0.001 | < 0.001 |
| Total score | 13.22 | < 0.001 | 37.70 | < 0.001 | 23.42 | < 0.001 | 64.3 | < 0.001 | 0.007 | < 0.001 |

BASDAI-Bath Ankylosing Spondylitis Disease Activity Index. Values are expressed as mean ± SD. P < 0.05 is considered as statistically significant.

**Supplementary Table S3 (a)**: Mean ± SD and P value of NDI questionnaire upon intragroup comparison

| Parameters | Groups | Mean ± SD | | | Intragroup comparison | | | |
| --- | --- | --- | --- | --- | --- | --- | --- | --- |
|  |  |  |  |  | Day 1 vs Day 14 | | Day 1 vs Day 28 | |
|  |  | Day1 | Day14 | Day28 | % difference | P value | % difference | P value |
| Pain intensity | Placebo | 3.08 ± 0.71 | 2.87 ± 0.53 | 2.86 ± 0.54 | 6.82 | 0.203 | 7.14 | 0.096 |
|  | F-BSE | 2.80 ± 0.57 | 2.32 ± 0.55 | 1.80 ± 0.70 | 17.14 | 0.001 | 35.71 | < 0.001 |
|  | C-BSE | 2.79 ± 0.58 | 1.95 ± 0.62 | 1.41 ± 0.50 | 30.11 | < 0.001 | 49.46 | < 0.001 |
| Difficulty in reading | Placebo | 2.29 ± 0.55 | 2.12 ± 0.53 | 2.04 ± 0.56 | 7.42 | 0.295 | 10.92 | 0.171 |
|  | F-BSE | 2.24 ± 0.52 | 1.80 ± 0.57 | 1.28 ± 0.73 | 19.64 | 0.009 | 42.86 | < 0.001 |
|  | C-BSE | 2.16 ± 0.63 | 1.75 ± 0.73 | 0.95 ± 0.55 | 18.98 | 0.057 | 56.02 | < 0.001 |
| Difficulty in driving | Placebo | 1.83 ± 0.48 | 1.66 ± 0.56 | 1.69 ± 0.55 | 6.28 | 0.213 | 9.42 | 0.257 |
|  | F-BSE | 1.84 ± 0.47 | 1.32 ± 0.55 | 1.12 ± 0.43 | 28.26 | < 0.001 | 39.13 | < 0.001 |
|  | C-BSE | 1.87 ± 0.53 | 1.29 ± 0.55 | 0.83 ± 0.56 | 31.02 | < 0.001 | 55.61 | < 0.001 |
| Difficulty in work | Placebo | 1.91 ± 0.40 | 1.79 ± 0.58 | 1.73 ± 0.54 | 9.29 | 0.328 | 7.65 | 0.257 |
|  | F-BSE | 1.92 ± 0.57 | 1.36 ± 0.63 | 1.24 ± 0.52 | 29.17 | 0.008 | 35.42 | < 0.001 |
|  | C-BSE | 1.95 ± 0.55 | 1.33 ± 0.70 | 0.95 ± 0.55 | 31.79 | 0.002 | 51.28 | < 0.001 |
| Total score | Placebo | 18.79 ± 2.24 | 18.04 ± 1.94 | 18.13 ± 1.71 | 7.26 | 0.137 | 7.82 | 0.182 |
|  | F-BSE | 18.48 ± 3.12 | 13.76 ± 2.43 | 11.12 ± 3.67 | 25.54 | < 0.001 | 39.83 | < 0.001 |
|  | C-BSE | 18.87 ± 3.79 | 12.83 ± 2.76 | 8.91 ± 2.08 | 32.01 | < 0.001 | 52.78 | < 0.001 |

NDI- Neck Disability Index. Values are expressed as mean ± SD. P < 0.05 is considered as statistically significant.

**Supplementary Table S3 (b)**: Percentage change and P value of NDI questionnaire upon intergroup comparison

| Parameters | Placebo vs F-BSE | | | | Placebo vs C-BSE | | | | F-BSE vs C-BSE | |
| --- | --- | --- | --- | --- | --- | --- | --- | --- | --- | --- |
|  | Day14 | | Day 28 | | Day14 | | Day 28 | | Day14 | Day 28 |
|  | % difference | P value | % difference | P value | % difference | P value | % difference | P value | P value | P value |
| Pain intensity | 19.16 | < 0.001 | 37.06 | < 0.001 | 32.06 | < 0.001 | 50.70 | < 0.001 | 0.133 | 0.034 |
| Difficulty in reading | 15.09 | 0.047 | 37.25 | < 0.001 | 17.45 | 0.050 | 53.43 | < 0.001 | 0.505 | 0.951 |
| Difficulty in driving | 20.48 | 0.036 | 33.73 | < 0.001 | 22.29 | 0.024 | 50.89 | < 0.001 | 0.528 | 0.233 |
| Difficulty in work | 24.02 | 0.018 | 28.32 | 0.002 | 25.70 | 0.018 | 45.09 | < 0.001 | 0.230 | 0.810 |
| Total score | 23.73 | < 0.001 | 38.67 | < 0.001 | 28.88 | < 0.001 | 50.85 | < 0.001 | 0.852 | 0.421 |

NDI- Neck Disability Index. Values are expressed as mean ± SD. P < 0.05 is considered as statistically significant.

**Supplementary Table S4**: Mean ± SD, percentage change and P value of NLRP3 and IL-1β questionnaire upon intergroup comparison

NLRP3- nucleotide-binding domain, leucine-rich repeat, and pyrin domain-containing protein 3; IL-1β- Interleukin-1β. Mean ± SD and P value of NLRP3 and IL-1β upon intergroup comparison

| Parameter | Groups | Intragroup comparison | | | |  | Intergroup | | | | |
| --- | --- | --- | --- | --- | --- | --- | --- | --- | --- | --- | --- |
|  |  | Mean ± SD | | % difference | P value | Groups | Placebo vs F-BSE | | Placebo vs C-BSE | | F-BSE vs C-BSE |
|  |  |  |  |  |  |  | % difference | P value | % difference | P value | P value |
| NLRP3 | Placebo | 0.618 ± 0.12 | 0.562 ± 0.11 | 9.06 | 0.158 | NLRP3 | 23.49 | < 0.001 | 35.05 | < 0.001 | 0.111 |
|  | F-BSE | 0.611 ± 0.11 | 0.430 ± 0.11 | 29.62 | < 0.001 |  |  |  |  |  |  |
|  | C-BSE | 0.601 ± 0.20 | 0.365 ± 0.14 | 39.27 | < 0.001 |  |  |  |  |  |  |
| IL-1β | Placebo | 0.600 ± 0.77 | 0.562 ± 0.97 | 6.33 | 0.078 | IL-1β | 43.77 | < 0.001 | 54.63 | < 0.001 | 0.059 |
|  | F-BSE | 0.592 ± 0.11 | 0.316 ± 0.77 | 46.62 | < 0.001 |  |  |  |  |  |  |
|  | C-BSE | 0.627 ± 0.19 | 0.255 ± 0.12 | 59.33 | < 0.001 |  |  |  |  |  |  |
